# Reliability of Gradient-Echo Magnetic Resonance Elastography of Lumbar Muscles: Phantom and Clinical Studies

**DOI:** 10.3390/diagnostics12061385

**Published:** 2022-06-03

**Authors:** Tsyh-Jyi Hsieh, Ming-Chung Chou, Yi-Chu Chen, Yi-Chen Chou, Chien-Hung Lin, Clement Kuen-Huang Chen

**Affiliations:** 1 Department of Medical Imaging, Chi Mei Medical Center, Yongkang, Tainan 71004, Taiwan; tsyhjyi.hsieh@gmail.com (T.-J.H.); yichenchou@hotmail.com (Y.-C.C.); chienhung0822@gmail.com (C.-H.L.); ckhc821@gmail.com (C.K.-H.C.); 2 Department of Radiology, Faculty of Medicine, College of Medicine, Kaohsiung Medical University, Kaohsiung 80708, Taiwan; 3 Department of Medical Imaging and Radiological Sciences, Kaohsiung Medical University, Kaohsiung 80708, Taiwan; lucky0084282@gmail.com; 4 Center for Big Data Research, Kaohsiung Medical University, Kaohsiung 80708, Taiwan; 5 Department of Medical Research, Kaohsiung Medical University Hospital, Kaohsiung 80708, Taiwan

**Keywords:** MR elastography, stiffness, technical failure rate, quality score, muscle, lumbar spine

## Abstract

Magnetic resonance elastography (MRE) has been used to successfully characterize the mechanical behavior of healthy and diseased muscles, but no study has been performed to investigate the reliability of MRE on lumbar muscles. The objective of this work was to determine the reliability of MRE techniques on lumbar muscles in both ex vivo phantom and in vivo human studies. In this study, fresh porcine leg muscles were used in the phantom study, and 80 healthy adults (38.6 ± 11.2 years, 40 women) were recruited in the human study. Five repeated stiffness maps were obtained from both the phantom and human muscles by using a gradient-echo MRE sequence with a pneumatic vibration on a 1.5 T MR scanner. The technical failure rate, coefficient of variation (CV), and quality score were assessed to evaluate the reliability of MRE, respectively. Analysis of variance was performed to compare the stiffness between different lumbar muscles, and the difference was significant if *p* < 0.05 after Bonferroni correction. The results showed that the MRE achieved a zero technical failure rate and a low CV of stiffness (6.24 ± 1.41%) in the phantom muscles. However, in the human study, the MRE exhibited high CVs of stiffness (21.57%–25.24%) in the lumbar muscles, and the technical failure rate was higher in psoas muscles (60.0–66.3% in) than in paraspinal muscles (0.0–2.5%). Further, higher quality scores were noticed in paraspinal muscles (7.31–7.71) than those in psoas muscles (1.83–2.06). In conclusion, the MRE was a reliable technique to investigate the mechanical property of lumbar muscles, but it was less reliable to assess stiffness in psoas muscles than paraspinal muscles.

## 1. Introduction

The lumbar muscles, including the paraspinal muscles (multifidus and erector spinae), psoas, quadratus lumborum, interspinales, intertransversarii, and short rotator muscles, are important for the core stability and strengthening of the lumbar spine [1]. Impairment of the lumbar muscles is one of the major causes of lower back pain and is also the leading cause of disability worldwide [2]. For evaluation of the etiology of lower back pain, magnetic resonance (MR) imaging has been recognized as the most accurate image modality of choice for the detection of structural abnormalities of the disc, vertebrae, nerve root, facet joint, and surrounding muscles [3]. Although some previous studies reported that the cross-sectional area and fat infiltration of paraspinal muscles in MR imaging may be predictive of lower back pain, the associations between lower back pain and anatomic changes in MR imaging were inconsistent [4,5].

For functional evaluation of muscles, previous studies have demonstrated that both ultrasonography and MR elastography (MRE) were capable of characterizing the mechanical behavior of both healthy and diseased muscles [6,7,8,9,10,11,12,13,14,15,16,17,18]. In addition, MRE was shown to successfully assess the tissue stiffness in vivo, and the stiffness measured by the MRE imaging technique has been confirmed as the value for the non-invasive assessment of liver fibrosis [19,20,21]. Recently, MRE was previously applied in the assessment of muscle stiffness, mostly on thigh muscles, and the results demonstrated that MRE was a sensitive technique to quantitatively detect changes in muscles [9,10,11,12,13,16,17,18,22].

However, in spinal muscles, the psoas muscles are situated next to the spinal column, whereas the paraspinal muscles are posterior to the spinal processes. When applying a pneumatic vibration on one’s lower back, the amplitude of vibration may be attenuated by the spinal processes, and hence the vibrating wave may not be properly generated in psoas muscles. To date, although some previous studies investigated the application of MRE on lumbar muscles [16,23], the reliability of MRE in lumbar muscles has not been well discussed. It is necessary to understand whether the MRE technique is clinically reliable for measuring stiffness in lumbar muscles.

In this study, we hypothesized that MRE may have a higher technical failure rate and lower image quality in psoas muscles than paraspinal muscles. Therefore, the purpose of this study was to evaluate the reliability of MRE, including technical failure rate, coefficient of variation (CV), and quality score, using both ex vivo porcine muscles and in vivo human lumbar muscles.

## 2. Materials and Methods

### 2.1. Phantom Study

**Figure 1:**
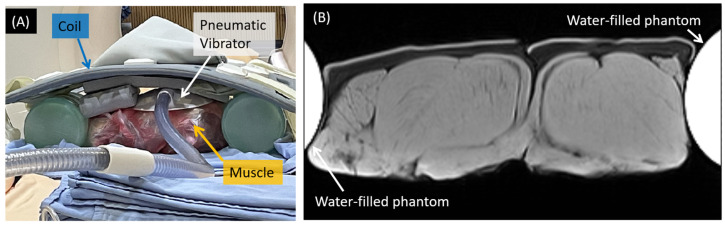
The ex vivo muscle phantom. (**A**) Two pieces of muscles were immobilized between two water-filled phantoms. A pneumatic vibrator was placed above the muscle, and a phased array coil on the topmost was used for signal receiving. (**B**) A cross-sectional T1-weighted image of the porcine muscles.

To understand the reliability of MRE in muscles, two pieces of fresh porcine muscles were wrapped together in a plastic bag and were immobilized on a table between two water-filled phantoms, as shown in Figure 1. The cross-sectional areas of each muscle were 145.7 ± 43.6 cm^2^, measured from MR images.

### 2.2. Human Subjects

This prospective single-center study was approved by our local institutional review board (IRB Serial No.: 10908-001), and written informed consent was obtained from each participant. Between August 2020 and March 2021, 80 healthy adults aged between 20 and 60 years were recruited. To avoid possible confounding factors, participants with sciatica, significant scoliosis, recent lower back injury, and surgery at the spine or lower back soft tissue were excluded from the study. In addition, those who had claustrophobia and pregnancy were excluded from the study.

### 2.3. MRI Acquisition

**Figure 2:**
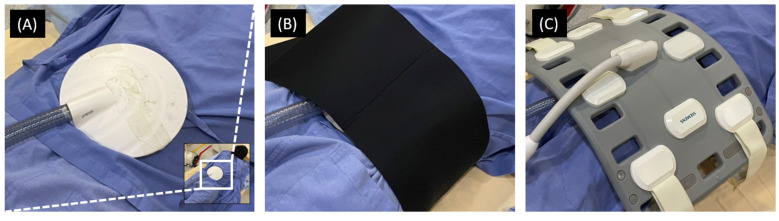
The preparation for the MRE scan of human lumbar muscles. (**A**) A subject lay on the table in a prone position, and a pneumatic vibrator was placed on his lower back. (**B**) An elastic strap was used to immobilize the vibrator by wrapping around the subject. (**C**) A phased array coil was placed on the topmost for signal receiving.

The MRE acquisition was performed on a 1.5 T MR scanner (MAGNETOM Aera; Siemens Healthcare, Erlangen, Germany) with an 18-channel phased-array body matrix coil. In the phantom study, the porcine muscles were positioned with fiber orientations parallel to the magnetic field, and a pneumatic vibrator was placed on its top. In the human study, subjects were scanned in a prone position with a pneumatic vibrator placed on their lower back, at the level of the lumbar spine, as shown in Figure 2. After tri-planar localizer scans were performed, T1-weighted gradient-echo DIXON pulse sequence was acquired (TR/TE = 6.69/2.39 ms, flip angle = 10 degrees, echo-train-length = 2, field-of-view = 345 × 380 mm^2^, matrix size = 290 × 320, slice thickness = 4 mm, number of slice = 36, and number of excitation = 1). Subsequently, MRE was performed using gradient echo pulse sequence (TR/TE = 50/21.34 ms, flip angle = 20 degrees, field-of-view = 360 × 360 mm^2^, matrix size = 128 × 128, number of phase = 4, slice thickness = 5 mm, number of excitation = 1) and pneumatic vibration (frequency = 100 Hz) generated by the manufacturer-supplied devices (Resoundant, Rochester, MN, USA). The MRE was repeated five times for statistical analysis.

### 2.4. Image Processing

In MRE quantification, the shear modulus for stiffness (kPa) was estimated using four different phase images, and stiffness maps with and without 95% confidence intervals (CI) were obtained using commercial software in Siemens workstation. In the 95% confidence map, the stiffness values with lower confidence levels were removed to keep good data quality [24,25].

**Figure 3:**
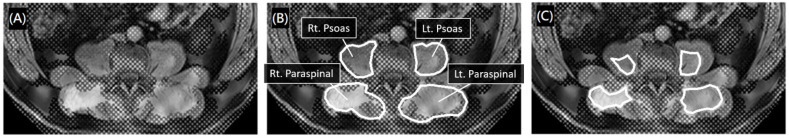
A 51-year-old male subject. (**A**) A fusion image of the T1-weighted DIXON water-only image. The post-calibration confidence map showed large regions (>50% of muscle size) with acceptable 95% confidence at bilateral paraspinal muscles and bilateral psoas muscles. The hashed areas indicate confidence index <95%. (**B**) The mean stiffness values were measured by four ROIs covering the entire bilateral paraspinal and psoas muscles, respectively. (**C**) The mean stiffness values were measured by four ROIs covering regions with 95% confidence index by excluding hashed areas.

To quantify the stiffness, a manual region-of-interest (ROI) analysis was performed by placing ROIs on bilateral psoas muscles and paraspinal muscles (including erector spinae and multifidus muscles), respectively, as shown in Figure 3. The mean values of stiffness were measured for each muscle, respectively. In addition, a CV defined as the standard deviation divided by the mean (%) was calculated on a voxel-by-voxel basis to understand the reproducibility of stiffness in five repeated scans, and mean CVs were calculated for statistical analysis. Moreover, another ROI analysis was performed on the 95% CI of the stiffness map by excluding those pixels with a low confidence index, as shown in Figure 3C.

### 2.5. Reliability of MRE

To evaluate the reliability of the MRE technique, the technical failure was defined as no pixel with a confidence index higher than 95% on the confidence map according to the previous studies [24,25]. When a stiffness map of MRE showed no pixel with a confidence index >95% within the muscle of interest, the stiffness could not be measured accurately from the muscle and the MRE scan was regarded as a technical failure. In the present study, the technical failure was determined for each scan by a senior musculoskeletal radiologist (T.J.H). Second, the data quality score was recorded for each scan, as follows: 0: no pixel with a confidence higher than 95%; 1: confidence map covering an area approximately less than 25% of the target muscle; 2: confidence map covering an area approximately between 25% and 50% of the target muscle, and 3: confidence map covering an area approximately more than 50% of the target muscle. An overall image quality score of five repeated scans was computed by summing up the data quality score of each scan (max score = 15).

### 2.6. Statistical Analysis

Data were presented as mean, standard deviation, and 95% CI. A Student’s *t*-test was performed to examine the effect of sex on the variables of interest, and the results were considered statistically significant if *p* < 0.05. The stiffness values of four lumbar muscles were compared using one-way analysis of variance (ANOVA), and the results were considered statistically significant if *p* < 0.05 after Bonferroni correction. All statistical analysis was carried out using IBM SPSS statistical software (Statistical Package for Social Science, IBM Corporation, New York, NY, USA) version 20.

## 3. Results

### 3.1. Phantom Study

**Figure 4:**
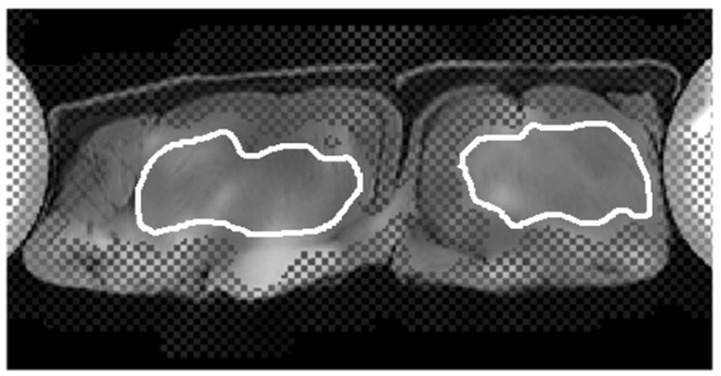
A fusion image of the T1-weighted DIXON water-only image. The post-calibration confidence map showed large regions (>50% of muscle size) with acceptable confidence in the two muscles, and two ROIs were placed on the two muscles for stiffness measurement.

The stiffness of porcine muscles was measured from eight different regions in four consecutive slices (two ROIs per slice), as shown in Figure 4. The mean stiffness value was 3.97 ± 1.07 kPa (95% CI: 3.08 kPa, 4.87 kPa), and the mean CV was 6.24 ± 1.41% (95% CI: 5.06%, 7.41%). In the 95% confidence map, the mean stiffness value was 4.13 ± 1.13 kPa (95% CI: 3.19 kPa, 5.08 kPa), and the mean CV was 6.19 ± 2.39% (95% CI: 4.19%, 8.19%). In the reliability test, the technical failure rate of the MRE in the phantom images was 0, and the overall image quality score was 15 for each region.

### 3.2. Human Subjects

The data available for analysis were from 80 subjects (mean age, 38.6 ± 11.2 years; range, 21–60 years): 40 women (mean age, 38.4 ± 11.4 years; range, 21–58 years) and 40 men (mean age, 39.4 ± 11.2 years; range, 21–60 years). The mean values of stiffness in the psoas and paraspinal muscles with and without 95% confidence maps are presented in Table 1. The results showed that the females exhibited statistically significantly higher tissue stiffness than males in bilateral entire psoas muscles (right: *p* = 0.005; left: *p* = 0.006). In the 95% confidence map, the results also demonstrated that females had statistically significantly higher stiffness than males in the left paraspinal muscle (*p* = 0.024) and right psoas muscle (*p* = 0.025).

In the reliability test, the technical failure rates of the MRE imaging technique were statistically significantly higher at the psoas muscles than at the paraspinal muscles in both the scan-based data (mean: 39.1% (625/1600); right paraspinal muscle: 7.0% (28/400); left paraspinal muscle: 8.3% (33/400); right psoas muscle: 67.3% (269/400); left psoas muscle: 73.8% (295/400); paraspinal muscles vs. psoas muscles, all *p* < 0.0001) and the subject-based data (technical failure in five repeated scans; mean; 32.2% (103/320); right paraspinal muscle: 0.0% (0/80); left paraspinal muscle: 2.5% (2/80); right psoas muscle: 60.0% (48/80) subjects; left psoas muscle: 66.3% (53/80); paraspinal muscles vs. psoas muscles, all *p* < 0.0001).

In the reproducibility test, the mean CVs measured in the psoas and paraspinal muscles are presented in Table 2. The mean CVs of females were statistically significantly higher than that of males at bilateral paraspinal muscles and bilateral psoas muscle (all *p* < 0.05). In addition, the mean CVs of bilateral psoas muscles were lower than those at bilateral paraspinal muscles, and there were statistically significant differences in the mean CVs between left psoas muscle and bilateral paraspinal muscles (right: *p* = 0.0002; left: *p* = 0.0007).

**Figure 5:**
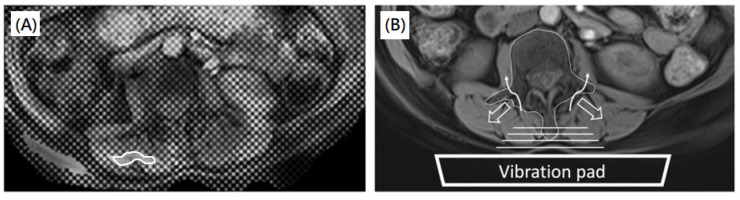
A 55-year-old female subject. (**A**) A fusion image of the T1-weighted DIXON water-only image. The post-calibration 95% confidence stiffness map showed a small region with acceptable confidence only at the right paraspinal muscle, as indicated by a white ROI. (**B**) The schematic diagram showed the possible influence by the osseous spine. The hallow arrows indicated the possible reflection waves due to the osseous spine, whereas the curve arrows indicated the partial vibration waves through the osseous spine.

**Figure 6:**
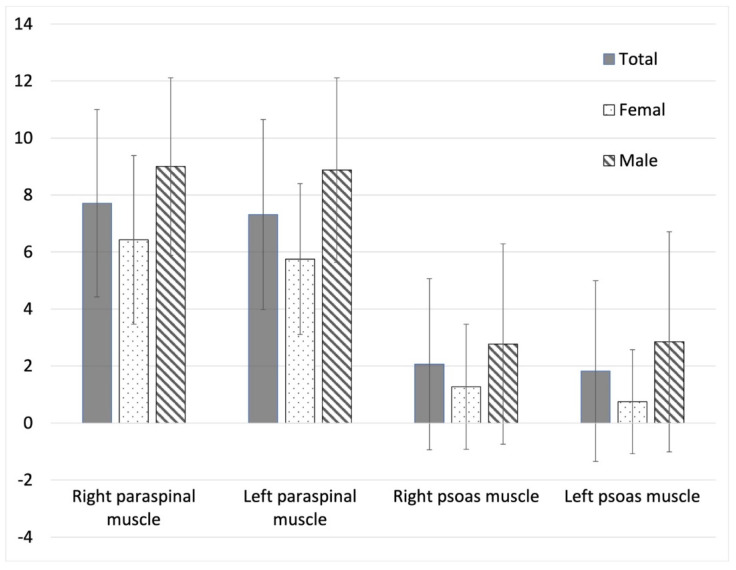
The quality score of the MRE based on 95% confidence map at paraspinal and psoas muscles.

In 71.4% of scans, the sizes of areas within the 95% confidence stiffness map were smaller than 25% of the sizes of the target muscles, as shown in Figure 5. The mean quality score was 4.71 ± 3.20 (0–15) in all lumbar muscles of all enrolled subjects, as shown in Figure 6. The mean quality scores of paraspinal muscles (right paraspinal muscles: 7.71 ± 3.29 (1–15); left paraspinal muscle: 7.31 ± 3.33 (0–15)) were significantly higher than those of psoas muscles (right psoas muscle: 2.06 ± 3.00 (0–11); left psoas muscle: 1.83 ± 3.17 (0–15); all *p* < 0.0001). In addition, the mean quality scores in females were significantly lower than those in males for all lumbar muscles (right paraspinal muscles: 6.43 ± 2.96 (1–13) vs. 9.00 ± 3.29 (4–15), *p* = 0.003; left paraspinal muscle: 5.75 ± 2.65 (0–11) vs. 8.88 ± 3.24 (3–15), *p* < 0.0001; right psoas muscle: 1.28 ± 2.20 (0–8) vs. 2.78 ± 3.51 (0–11), *p* = 0.0252; left psoas muscle: 0.75 ± 1.82 (0–9) vs., 2.85 ± 3.86 (0–15), *p* = 0.0029).

## 4. Discussion

To the best of our knowledge, this is the first study to assess the reliability of the MRE technique using both ex vivo porcine muscles and in vivo human lumbar muscles. The results demonstrated that MRE had excellent reliability with zero technique failure rate and good inter-scan reproducibility (CV < 7%) in the porcine muscles. However, the mean technical failure rate of MRE was as high as 39.4% (0.0–2.5% in paraspinal muscles; 60.0–66.3% in psoas muscles), and mean CVs for stiffness were between 21.57% and 25.24% in human lumbar muscles. In 71.4% of scans, the areas within 95% confidence were smaller than 25% of the target muscles. Overall, the findings suggest that the reliability of the MRE technique was high for measuring the stiffness in the porcine muscle, moderate in human paraspinal muscles, and low in human psoas muscles.

MRE is regarded as a highly accurate and reliable non-invasive diagnostic technique for the detection and staging of liver fibrosis with a low technique failure rate (approximately 5–6%) and good reproducibility (CV < 10%) [24,25,26]. However, the present study showed that the MRE had high technical failure rates and low reproducibility (CV > 20%) in human lumbar muscles. Although many previous studies have demonstrated that MRE was feasible to measure muscle stiffness, the technical failure rate and reproducibility of the MRE technique have not been well discussed. One previous study has demonstrated that the MRE had a low poor-quality rate of 2.9% (2/68, failure to measure stiffness in 95% confidence map) at paraspinal muscles [23]. Consistently, our findings showed that the technical failure rate was as low as 1.3% at paraspinal muscles. However, the technical failure rate was higher than 60% at psoas muscles, suggesting that MRE was less reliable to assess the stiffness in psoas muscles than paraspinal muscles.

In line with a previous study [27], our results showed that the MRE exhibited good reproducibility in an ex vivo muscle study with CVs < 7%, indicating that the manufacturer-supplied devices and MRE pulse sequence were reliable in the assessment in muscle stiffness. In the human studies, a previous study showed that the reproducibility was variable at different muscles of the same thighs (CV: 5.3–21.9%) [12]. However, our subject study showed that the reproducibility of MRE was low (CV: 21.6–25.2%) in both paraspinal and psoas muscles. It was previously shown that the stiffness values in lumbar muscles were varied by changing the locations of the vibration pad [16]. The lower CVs of stiffness in the lumbar muscles speculate that the vibration was significantly influenced by the osseous spine. In paraspinal muscles, the shear wave was induced mainly by ipsilateral vibration and partially from the vibration of the contralateral side through the lumbar spine. Even though the vibration pad was placed at the center of the lower back, the waves were found uneven in bilateral paraspinal muscles. This uneven phenomenon may be attributable to the irregular wave reflection caused by a concave interface formed from the osseous spine, including spinous process, lamina, and transverse process (Figure 5B).

Furthermore, the MRE technique had higher technical failure rates (up to 67.3–73.8%) and lower mean quality scores at the psoas muscles than those of paraspinal muscles. The findings were likely attributable to the indirect vibration, susceptibility, and motion artifacts. First, a previous study demonstrated that mean oscillation amplitude in psoas muscles was significantly lower than that in paraspinal muscles [16]. The authors proposed that the major source of vibration at the psoas muscles was from the indirect vibration, whereas the major source of vibration at paraspinal muscles was from the direct vibration [16]. As the source of vibration at psoas muscles may come from both direct and indirect vibrations, the mixed waves may lead to high technical failure rates, low quality scores, and low reproducibility at the psoas muscles. Second, it was previously shown that the technical failure rates of gradient-echo MRE were higher at 3.0 T than 1.5 T, due likely to more susceptibility artifacts (i.e., more signal loss) and lower quality of wave imaging at higher field strength [24]. In the present study, both psoas and paraspinal muscles were adjacent to the spine and had more susceptibility artifacts, so the technical failure rates were higher in the spinal muscles than the porcine muscle. In addition, the psoas muscles are situated between the spinal column and abdominal organs, and likely suffered from more susceptibility artifacts, leading to higher technical failure rates than the paraspinal muscles. Third, the present study measured muscle stiffness using gradient-echo-based MRE, which was demonstrated to require a longer scan time than spin-echo echo-planar imaging (EPI)-based MRE [28]. Previous liver studies showed that both techniques had excellent inter-observer agreement for measured liver stiffness, but the gradient-echo MRE exhibited more breathing artifacts and higher technical failure rates than EPI MRE due likely to its longer scan time [28,29]. As the psoas muscles were close to the abdominal organs and prone to motion, the higher technical failure rate and lower quality score at the psoas muscles than at the paraspinal muscles were partly attributable to the breathing artifacts in the gradient-echo MRE. Taken together, the higher technical failure rates in the psoas muscle than paraspinal muscles were likely associated with the indirect vibration, susceptibility artifacts, and motion artifacts when using the gradient-echo MRE technique. Further research is needed to evaluate the feasibility of EPI-based MRE in psoas muscles.

In sex differences, some previous studies demonstrated that there was a sex difference in muscle stiffness based on ultrasound shear wave elastography, and that males generally had higher muscle stiffness than females [30,31,32]. However, the sex differences in muscle stiffness have not been previously evaluated for paraspinal and psoas muscles using the MRE technique. In the present study, our results of the MRE technique showed that females had significantly higher muscle stiffness than males at the left paraspinal muscles and right psoas muscles according to the MRE with a 95% confidence map and there was no significant difference in other muscles. The inconsistent results between our study and previous ultrasound studies may be associated with a high technique failure rate and low reproducibility of MRE, especially in psoas muscles. Further research is needed to confirm the sex differences of stiffness in lumbar muscles.

Our study had some limitations that warrant discussion. First, most of the participants in our study were young, so the relationship between stiffness of lumbar muscles and age could not be well discussed. Second, the sample size was small, and the enrolled subjects were healthy volunteers without significant spine diseases. Third, we did not evaluate the core muscle function in each subject, so the correlation between muscle stiffness and muscle function could not be clarified.

## 5. Conclusions

In conclusion, our results confirmed that the MRE technical failure rate was lower in paraspinal muscles than in psoas muscles. The higher technical failure rate, higher CVs, and lower quality score in psoas muscles than those in paraspinal muscles were likely attributable to the geometry of osseous spine as well as possible breathing artifacts. Therefore, we concluded that gradient-echo-based MRE technique was clinically feasible for studying stiffness of paraspinal muscles in clinical setting, but it was less reliable for psoas muscles.

## Figures and Tables

**Table 1:** The mean stiffness measured from the entire muscle and in 95% confidence map for paraspinal and psoas muscles.

	Total	Female	Male	
Stiffness (kPa) in the entire muscle	n Mean ± SD (95% CI)	n Mean ± SD (95% CI)	n Mean ± SD (95% CI)	*p*
Right paraspinal muscle	80 1.95 ± 0.35 (1.87, 2.03)	40 1.98 ± 0.41 (1.85, 2.11)	40 1.92 ± 0.28 (1.83, 2.01)	0.442
Left paraspinal muscle	80 2.01 ± 0.38 (1.93, 2.10)	40 2.08 ± 0.47 (1.93, 2.23)	40 1.94 ± 0.23 (1.87, 2.01)	0.934
Right psoas muscle	80 1.60 ± 0.32 (1.53, 1.67)	40 1.70 ± 0.28 (1.60, 1.79)	40 1.50 ± 0.32 (1.40, 1.60)	0.005 *
Left psoas muscle	80 1.50 ± 0.30 (1.43, 1.57)	40 1.59 ± 0.23 (1.52, 1.66)	40 1.41 ± 0.34 (1.30, 1.52)	0.006 *
Stiffness in 95% confidence (kPa)				
Right paraspinal muscle	80 2.09 ± 0.52 (1.97, 2.21)	40 2.15 ± 0.56 (1.97, 2.33)	40 2.03 ± 0.48 (1.88, 2.18)	0.310
Left paraspinal muscle	78 2.22 ± 0.79 (2.04, 2.40)	38 2.43 ± 1.01 (2.10, 2.76)	40 2.02 ± 0.44 (1.88, 2.16)	0.024 *
Right psoas muscle	32 1.79 ± 0.43 (1.64, 1.95)	12 2.05 ± 0.52 (1.72, 2.37)	20 1.64 ± 0.28 (1.51, 1.77)	0.025 *
Left psoas muscle	27 1.74 ± 0.48 (1.55, 1.93)	8 1.80 ± 0.47 (1.41, 2.20)	19 1.71 ± 0.49 (1.48, 1.95)	0.661

* *p* < 0.05 indicates a significant difference between males and females.

**Table 2:** Mean CVs of stiffness at paraspinal and psoas muscles in MRE.

	Total (*n* = 80)	Female (*n* = 40)	Male (*n* = 40)	
CV (%)	Mean ± SD (95% CI)	Mean ± SD (95% CI)	Mean ± SD (95% CI)	*p*
Right paraspinal muscle	23.32 ± 7.17 (21.71, 24.92)	26.10 ± 6.73 (23.92, 28.28)	20.60 ± 6.59 (18.50, 22.71)	<0.001 *
Left paraspinal muscle	25.24 ± 6.95 (23.68, 26.79)	27.29 ± 6.96 (25.03, 29.54)	23.24 ± 6.42 (21.19, 25.29)	0.009 *
Right psoas muscle	21.57 ± 4.83 (20.49, 22.65)	22.66 ± 4.45 (21.22, 24.11)	20.50 ± 4.99 (18.90, 22.10)	0.045 *
Left psoas muscle	21.95 ± 4.82 (20.86, 23.03)	23.12 ± 4.87 (21.54, 24.70)	20.80 ± 4.55 (19.35, 22.26)	0.032 *

* *p* < 0.05 indicates a significant difference between males and females.

## Data Availability

Not applicable.
